# Evaluating programs for young people with a family member with mental health challenges: protocol for a mixed methods, longitudinal, collaborative evaluation

**DOI:** 10.1186/s40359-023-01104-7

**Published:** 2023-03-10

**Authors:** Andrea Reupert, Nerelie Freeman, Rochelle Hine, Sophie Lea, Nivedita Nandakumar, Charlotte O’Grady, Lefteris Patlamazoglou, Laura Pettenuzzo, Kim Foster

**Affiliations:** 1 School of Educational Psychology and Counselling, Faculty of Education, Monash University, 19 Ancora Imparo Way, 3800 Clayton, Australia; 2 Monash Rural Health, Monash University, 3820 Warragul, Australia; 3 The Satellite Foundation, 22 Easey St, 3066 Collingwood, Australia; 4 School of Nursing, Midwifery & Paramedicine, Australian Catholic University, Melbourne, VIC Australia

**Keywords:** Evaluation, Mixed methods, Participatory; parental mental illness, Youth mental health

## Abstract

**Background:**

Young people with a sibling or parent who experiences mental health challenges have their own support needs. Most programs designed for this population lack a strong evidence base, and the involvement of young people in the development and evaluation of programs designed to support them is unclear or lacking.

**Methods:**

This paper describes a protocol for a mixed methods, longitudinal, collaborative evaluation of a suite of programs delivered by The Satellite Foundation, a not-for-profit organisation for young people (5–25 years) who have a family member with mental health challenges. Young people’s lived experience and knowledge will guide the research approach. Institutional ethics approval has been obtained. Over a three year period, approximately 150 young people will be surveyed online on various wellbeing outcome measures, prior to, six and twelve months following program participation with data analysed using multi-level modelling. Groups of young people will be interviewed after participating in different Satellite programs each year. An additional group of young people will be interviewed individually over time. Transcripts will be analysed using thematic analysis. Young people’s creative artworks on their experiences will be included as part of the evaluation data.

**Discussion:**

This novel, collaborative evaluation will provide vital evidence on young people’s experiences and outcomes during their time with Satellite. Findings will be used to inform future program development and policy. The approach used here may guide other researchers engaging in collaborative evaluations with community organisations.

## Background

This protocol paper describes the mixed methods design and collaborative processes for a three-year external evaluation of a not-for-profit organisation, The Satellite Foundation (hereafter known as Satellite). Satellite delivers programs for children and young people aged 5–25 years who have a parent and/or sibling experiencing mental health challenges. Over a three-year period, the evaluation findings will be used to iteratively inform and further develop Satellite services. In addition to the research methods, this paper outlines steps to actively and authentically involve children and young people in a collaborative evaluation design and delivery process while simultaneously presenting meaningful insights for the funding body.

Governments need evidence that the expected results of social service initiatives they fund are achieved to support quality improvement, risk management, accountability, decision-making and ongoing funding. Addressing this need requires evaluations that employ robust research methodologies and use validated outcome tools, conducted by an independent or external team. Although participatory research is increasingly being used in psychological research [1], tensions may arise when employing this approach with young people, who may face barriers to participation and may have different or opposing views and preferences for the evaluation process.

Young people with a family member experiencing mental health challenges have diverse experiences and outcomes. Research shows that these children can experience associative stigma, caring responsibilities and stress from witnessing and responding to their relative’s illness [2, 3]. Young people whose parent/s has/have mental health challenges describe being marginalised [4], are more likely than their peers to develop a mental illness [5], report higher rates of injuries and health difficulties such as asthma [6], and present with educational, social and emotional impairments [7]. Likewise, several studies have found that those who have a sibling with mental health challenges are at risk for lower psychological functioning which can lead to mental health challenges [8]. The COVID-19 pandemic and lockdowns have added further layers of complexity for these young people, including isolation and stress [9]. As nearly 50% of mental health concerns begin in childhood before the age of 14 [10], and 21–23% of children grow up with a parent with a mental illness [11], developing and delivering evidence-based interventions to these young people is a public health priority.

It is important to recognise that while children can experience adverse psychological or mental health outcomes in these family contexts, not all children do [12]. Positioning young people within a risk and vulnerability lens alone has been critiqued as failing to represent a holistic view of these children [13]. This limited perspective obscures their choice, agency, strengths and competencies. Many children derive positive benefits from undertaking caring roles and responsibilities such as increased empathy and compassion [14], the development of life and professional skills, and resilience, persistence and maturation [15].

Most interventions for young people with a family member who experiences mental health challenges employ a peer support approach, which aims to promote connectedness, wellbeing, adaptive coping and mental health literacy [12, 16]. Many of these face-to-face interventions (for example ON FIRE [17]) are relatively small in scope, although in recent years online peer support programs have been developed which have the potential to promote reach and access [18]. However, online approaches are in the early stages of development and are typically limited to young people aged 18–25 years. Additionally, most online and face-to-face programs in this field lack a strong evidence base [19] and the involvement of young people in the development of the programs and their evaluations is unclear or lacking.

### Participatory program development and evaluation

Engaging stakeholders in program development is critical to ensure that resulting programs are grounded in the preferences and needs of intended end users [20]. Being actively involved and heard in this process has the potential to promote autonomy and empowerment, especially for marginalised young people [21]. Engaging young people in program development and evaluation needs to acknowledge the intersectionality of young people who live in these families and appreciate inequalities that may impact participation [4].

Participatory research is an umbrella term used to describe approaches that share a philosophy of inclusivity and engagement with end-users and relevant stakeholders in the research process, including evaluations [22]. There are different frameworks of youth participation. Cargo and Mercer [22] argued that the specific steps undertaken in participatory research will vary depending on whether the aim is to promote self-determination, translate research into practice and/or promote social justice, equity and access.

The process of implementing participatory evaluations with young people is not always easy or, at times, authentically conducted since young peoples’ input is often mediated through the perspective of adults and influenced by associated power imbalances [23]. In a scoping review of 54 participatory projects, only 17 included young people on boards, 18 involved them in the preparation stage and 28 included them in data collection and/or analysis [21]. There are many reasons why young people may not be involved in program development, some of which include funding and ethical guidelines, which tend to stipulate that a detailed methodology and timetable be submitted and approved before young people can be involved. The current study addresses a number of these concerns by gaining ethics approval prior to engaging with young people directly, then modifying the protocol based on their input and including young people’s perspectives and recommendations throughout the entire study. The following section describes the participatory approach used in this study.

### The Satellite Foundation

The research team was awarded a three-year contract by the Victorian Department of Health to evaluate the programs offered by Satellite. Satellite is an Australian not-for-profit organisation that aims to promote the mental health and wellbeing of children and young people (aged 5–25) when a family member (parent/carer or sibling) experiences mental health challenges. By delivering a range of in-person and online programs, Satellite aims to foster strong connections between young people, their families and the wider community. Satellite intends to promote positive change in young people not only by offering specific programs and follow-up activities but also through their engagement with other young people in co-designing and co-leading their programs. Satellite staff put significant time and energy into staying in contact with young people outside program delivery (e.g., after finishing one program but before starting another) to keep them connected with Satellite, an activity they refer to as “linking programs”. They also deliver education programs to schools and community services about the needs of young people living in these families, intending to promote a coordinated, systems approach to supporting these young people. See Table 1 for an overview of Satellite programs. Though the programs vary in intensity (i.e. dose), approach and medium (e.g. weekend camps, Zoom meetings) the overall aims of Satellite are to promote the wellbeing, resilience and connectedness of young people, aged 5–25 years, who have a family member experiencing mental health challenges.

**Table 1:** Satellite programs

Programs	Participants’ Ages	Brief description	Possible active ingredient/s
It’s a Mad World	18+	An experimental, group devised project for graduates of Satellite programs to create an online showcase of different perspectives on mental health, merging lived experience, design and creativity with a passion for destigmatising mental illness	Peer support and connection based on lived experience, respite from carer responsibilities and creative activities
Create and connect	11–14	Facilitated by professional musicians, artists, peer facilitators, and Satellite staff, these workshops offer playful experiences that allow participants to explore collaboration while reinforcing connection. Workshops include music and song writing, photography and visual art including graffiti and meme-making, and art and craft	Peer support and connection based on lived experience, respite from carer responsibilities and creative activities
Satellite connect (includes retreat)	18–25	Structured peer support and peer development program that gives young people a space and place to be heard, to learn new skills, and to share their stories, as well as ways their unique lived experience can be harnessed positively to shape and influence the lives of younger children. This program expands, develops and supports Satellite’s network of peer leaders and facilitators to co-design and co-facilitate programs for children and young people living in families where a family member has a mental illness.	Peer support and connection based on lived experience, respite from carer responsibilities, creative activities and wellbeing education
Satellite Connect Youth	14–17	Similar to that of Satellite Connect, but with a greater focus on the experience of sharing participant’s stories, being heard and facilitating peer connection and wellbeing.	Peer support and connection based on lived experience, respite from carer responsibilities, creative activities and wellbeing education
At Home with Satellite	8–14	A program of online curated creative workshops delivered across the school holidays. Participants can choose from four curated workshops with each workshop having a 1- 1.5 h Zoom session. Each workshop had a creative focus that explored ideas around mental health and wellbeing, connections and strengths. The workshops covered photography, making superheroes, song writing.	Peer support and connection based on lived experience, respite from carer responsibilities and creative activities
Connecting activities	All	While not a program, connecting include engagements with children and young people after programs to ensure they still feel part of our community even when the program has finished. Connecting activities vary, and include family fun days, visits to local attractions, post-program catch-ups and end of Year celebrations	Connectedness to others

Satellite works in partnership *with* young people in recognition of their lived experience expertise, the rights of young people to exercise self-determination and choice, and to ensure that the programs offered are relevant to the young people they aim to serve. Subsequently, interventions delivered by Satellite are the product of co-creation and co-facilitation with young people. Given that youth participation is at the heart of what Satellite does, it was considered important for the evaluation team to employ a similar approach. Taking a participatory approach in the evaluation was also seen as a means of providing further opportunities for young people to be engaged in program design and delivery.

Simultaneously, we were cognisant that as external evaluators we needed to maintain an independent stance so that the resulting evaluation was credible and trustworthy. There are many ways of operationalising this independence. Some assume the empirical view that evaluators need to completely distance themselves from the program, rely exclusively on extant data, and avoid contact with program staff in order to maintain ‘objectivity’ [24]. At the other end of the continuum, others argue that employing a participatory evaluation model where the evaluation team actively work with the program staff and end-users can benefit organisational learning, inform change [25] promote self-determination [26], and incorporate multiple valid perspectives and interpretations of meaning. Marklewicz [27] pointed out that participatory models extend the use of evaluation for funding accountability, by providing a process that builds the capacity of program participants, developers and facilitators. Nonetheless, adopting a participatory evaluation approach that includes program participants (young people) needs to have clearly defined expectations and boundaries of involvement from the outset [27].

The project team were funded by the Department of Health, via Satellite, to undertake this evaluation, making the team’s independence, notwithstanding the participatory approach, even more critical. Being funded by the very service we were evaluating may be problematic [28] including feeling pressured to report results that favour Satellite and/or not publishing results that might reflect unfavourably on Satellite. To safeguard against this potential bias, six monthly meetings will be held with the Department of Health, who will provide oversight of the overall evaluation plan, including analysis and results. The funding agreement included that the methodology and results would made available so the evaluation could be judged on its validity and significance, external to the evaluation team. This allows for an assessment of whether appropriate variables are being measured, the analysis employed is appropriate and whether the data reported support the final conclusions reached [28]. Documenting the evaluation protocol also allows for the evaluation to be replicated in other programs. Finally, it should be noted that the evaluation process allows for results to inform continual improvements to Satellite’s programs. Rather than simply being provided with a report at the end of the three years, Satellite have committed to this process, which also indicates their openness to receiving evaluating data that might not necessarily be favourable.

The current protocol describes the collaborative approach being used in this project and aims to provide guidance to other researchers. We are not able to ‘bracket’ our own ideas about the evaluation design [29] as our contracted, predetermined approach had been approved (and funded) by the relevant government authority. Due to the timeline and process for the funding tender, we were unable to consult with young people in the initial evaluation design. As the approved tender design included seeking input from young people about the evaluation approach, immediately upon gaining the funding we sought to include young people in the design and conduct of the evaluation, including the interpretation and dissemination of findings.

**Fig. 1:**
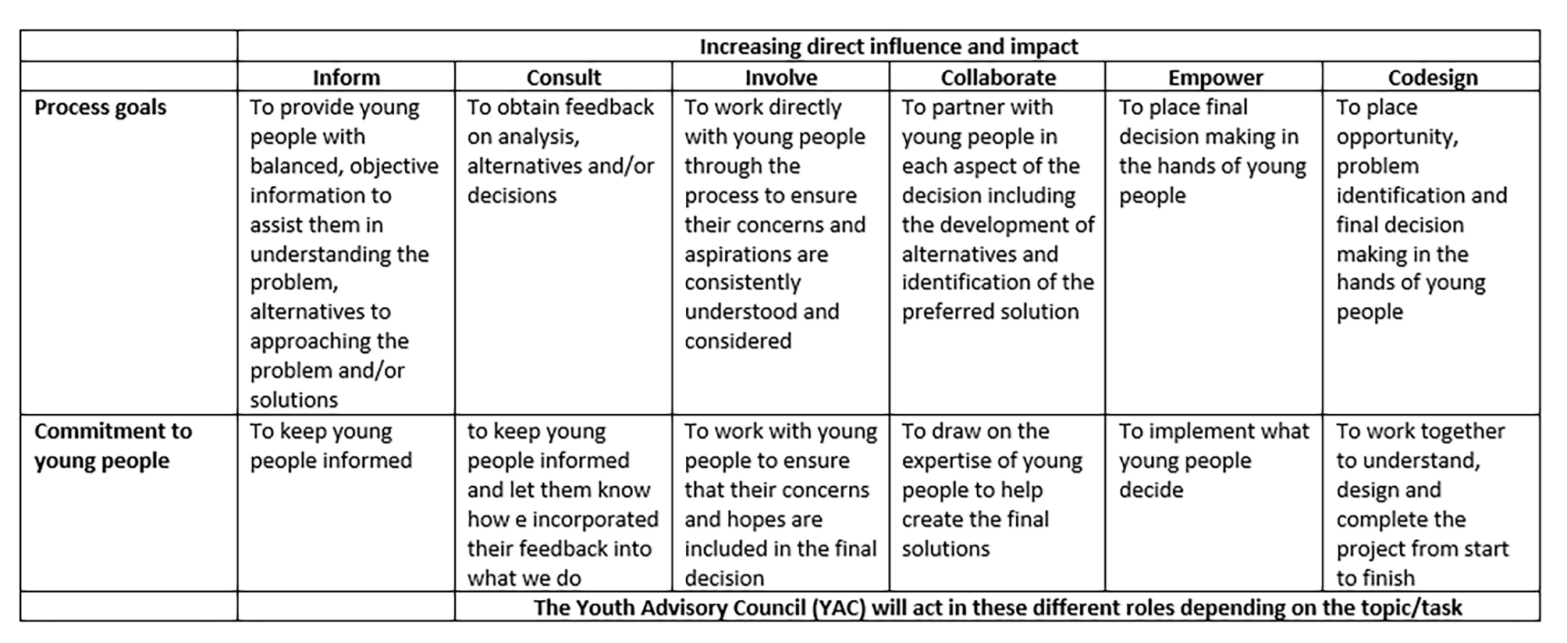
Satellite’s co-creation framework Adapted from [51, 52].

Our intent for working in this particular manner with young people was to ensure that the evaluation methodologies employed were inclusive, accessible and engaging, appropriate to the developmental competencies of young stakeholders, suited to their interests and, most importantly, promoted young people’s empowerment. Moreover, the evaluation design is underpinned by a similar philosophy/similar stance to working with young people to that of Satellite. The collaborative process presented builds on the experience of the authors in other community projects employing this design [30] and extends this work by focusing on the ways in which it can be built into an evaluation methodology. Adapted from other frameworks, Satellite developed a co-creation framework for their own delivery work, and asked the evaluation team to indicate where on the continuum of participation the evaluation participatory approach would sit (see Fig. 1). We have taken a “collaborate” stance in this project. Accordingly, the evaluation approach we will employ is multifaceted, with a range of opportunities for different groups of young people to collaborate. Although involving stakeholders in program development and evaluation has the potential to increase the relevance, usability, and credibility of the services and supports offered, the methods for doing so are not well understood nor often documented [20]. The process of writing this protocol in itself may promote a shared understanding of our participatory evaluation approach amongst the stakeholders involved (with young people from the Satellite advisory group being co-authors) and one which might offer guidance to others working in the field.

### Aim and objectives

The aim of this mixed methods, longitudinal participatory project is to evaluate the suite of programs delivered by the Satellite Foundation designed for young people (5–25 years) who have a family member with mental health challenges. Specific evaluation objectives are to:

- Explore the experiences of young people participating in Satellite programs,
- Identify the outcomes of young people participating in Satellite programs.

## Methods

This section identifies and describes the evaluation methodology employed and the role of stakeholders.

### Design

A mixed-methods, longitudinal, participatory design will be used. A mixed-methods approach with quantitative and qualitative data is appropriate for evaluations as it provides a range of evidence relevant to program objectives. Concurrent and sequential data collection will be used [31].

### Participatory stakeholder groups and roles

There are three stakeholder groups involved, each with distinctive though at times overlapping roles in the evaluation; the evaluation team, young people and Satellite staff. The evaluation team is comprised of university researchers who have individual and collective experience working with young people. Within the team, different staff are responsible for the various project components. The evaluation team leader takes overall responsibility for the project including oversight of the budget and ensuring that ethical processes are followed.

Satellite’s pre-existing Youth Advisory Council (YAC) will be the main way for young people to participate in the evaluation process. The YAC is comprised of 15 young people with lived experience of having family member/s with mental health challenges. The role of the YAC is to shape the work of Satellite by providing input and advice and co-creating programs. They are remunerated for their time by Satellite. Prospective YAC members apply for membership, and are selected by current YAC members, who ensure members have diverse backgrounds and varied exposure to Satellite (some with much, some with little or none).

To provide consistency and continuity, one evaluation team member (RH) will be the primary conduit between the YAC and the evaluation team. It will be their responsibility to work with and support the YAC throughout the evaluation design, interpretation and dissemination process. This team member brings substantial experience working with young people in a participatory manner. Additional evaluation team members will meet with the YAC as relevant throughout the three years. Other young people are involved in the evaluation as participants but also provide feedback on ongoing program development and the evaluation design (see below).

The final group of stakeholders are Satellite staff. They bring a close knowledge of Satellite; its vision aims and approaches, and are responsible for facilitating Satellite programs. Their participation is key to enhancing relevance, ownership and unitization of the findings to inform ongoing program development. Satellite staff will action the implications of the evaluation and make any relevant changes to program content and types.

### Initial participatory YAC workshop

A full-day workshop was held with the YAC at the beginning of the project. The day was facilitated by the evaluation team YAC contact (RH), Satellite’s Youth Liaison staff member, the Evaluation lead and co-lead, and other evaluation team members, and attended by the YAC and selected Satellite staff. The workshop was held at a location and at a time that made it easy for participants to attend. To accommodate participants’ capacities, priorities and preferences, including large and small group discussions, various pedagogies were used (e.g. Zoom and face-to-face; large and small group discussion). The language employed aimed to be age-appropriate, non-judgemental and jargon free, using the Mental Health Coordinating Council [32] guide for recovery-oriented language. Consideration of trauma-informed and inclusive principles were incorporated into the physical environment, with the aim of creating a safe and supportive space. To that end, a youth-designed community space was used, in which the sensory impact has been considered and modified (e.g. gentle music, soft lighting, furniture placement that enables connection). Similarly, participants had access to, and were aware of support options as required, with breaks offered as needed, in addition to scheduled meal breaks. See Table 2 for an outline of the workshop. The workshop concluded by inviting YAC participants to evaluate how inclusive the workshop was. Their collated and de-identified feedback were provided back to the YAC with action steps, in future deliberations with the Council.

**Table 2:** Youth Advisory Council: workshop details

Session title	Session aims	Session topics and process
Welcome and introductions	To set the scene for the day	• In a large group format: • Acknowledgement of country • Recognition and appreciation of lived experience of having a family member with mental health challenges • Introductions • Expectations of the day (e.g. right for others to speak/not to speak etc.) • Clarify participant roles (e.g. to speak for themselves but also represent other young people) • Present an overview of the day
What is program evaluation?	To gain an understanding of program evaluation Share experiences of being involved in evaluation To identify ways for promoting evaluation engagement	• Overview of what program evaluation is • In small groups, identify an experience of being involved in evaluation (or not being involved) e.g. in other research projects, in the community, retail outlets etc., and what was good/not so good about that process • Suggest ways to promote young people’s recruitment and engagement in evaluation
Evaluation outcomes	To identify what outcomes the evaluation should target and subsequently measure.	• Think-pair-share: Invite participants to respond to two questions: • How would you describe how your life has changed since being part of Satellite? • How do you think Satellite will change the lives of other young people? • Encourage participants to articulate changes in terms of measurable outcomes • Using an anonymous, online poll vote to identify the most important outcomes.
Interviews	To elicit the types of questions we should be asking and the language and approach for the interviews	• World café format in which participants work in small groups to respond to different questions: • What questions should we be asking for different age groups? • What language should we use for different age groups? • What approach should we use for different age groups?
Ongoing YAC involvement	To discuss YAC future involvement	• In the large group, discuss how the YAC can assist in: • Informing program development • Informing ongoing evaluation – are we asking the right questions? o How often to meet and for how long? • Conclusion, thanks and acknowledgement of all views
Evaluating the evaluators	To obtain feedback on the day	• Participants will be asked to complete an evaluation form asking: • what was good about the day • what could have been improved • on a Likert scale from 0, not at all, through to 10, very much so, how much they felt they had an opportunity to speak up and how much they felt heard.

The feedback and advice generated from the workshop was used to inform the evaluation approach outlined here. For example, the outcomes identified by YAC participants were used to identify relevant outcome measures. Likewise, their advice about interviewing was used when deciding on approaches (focus group or individual etc.) and the types of questions that should be asked.

### Ongoing YAC collaboration

During the three-year evaluation, we aim to meet every three months with the YAC to discuss evaluation results and ask for their feedback about (i) what the results might mean, (ii) the implication of the results for program development and (iii) appropriateness of the data collected, and what changes may be required to the evaluation process. Their views will be used to inform future rounds of the evaluation and Satellite offerings. Changes that were or were not actioned regarding Satellite programs, and why, will be discussed at subsequent YAC meetings, to ensure the evaluation and program development processes are transparent and accountable.

### Data collection

As per Table 1, Satellite offers a range of program activities, with varying lengths (e.g. a weekend camp or one-off event) and in different mediums (e.g. online, face to face). Additionally, many young people participate in more than one activity. Thus, rather than evaluating each program activity as a distinct entity, the evaluation needs to reflect the ongoing and ecological nature of all of Satellite’s offerings. Notwithstanding this, Satellite were also keen to delve deeper into some program activities which were considered to be representative of their vision and objectives. Using input from the YAC, the data collection method is outlined below.

### Semi-structured interviews or conversations

The YAC recommended these be called conversations rather than interviews, indicating that this term would be more acceptable to young people A purposive sampling approach will be used to conduct semi-structured face-to -face, telephone or Zoom individual conversations or focus groups with Satellite participants. Though there is some debate about the efficacy of these different mediums, some have argued that there is little difference in outcomes, depending on the research questions posed [33]. Conversations are based on a narrative approach which aims to illuminate the value of a program and highlight what needs to occur to improve it [34]. Each year, three programs will be identified by Satellite staff that they consider to be particularly important to their overall offerings and nine young people from each of those three programs will be invited to a conversation each year. The sample size of nine per program was chosen as it is considered adequate for sampling amongst a homogenous population while also allowing for deep analysis [35]. The YAC provided feedback on what should be asked and how. A key element of the evaluation approach is not only evaluating what Satellite does but also how successfully they work with young people in a participatory manner. Thus, questions will be asked, in an age appropriate way, about how involved the young person was in the program/s they were a part of, and whether they wanted to be more/less involved. Other questions will be around process (e.g. what parts of the program were important to you?), accessibility (e.g. how easy was it to get to the program?), safety (e.g. how safe did you feel participating in the program) and outcomes (e.g. what changed, if anything, for you, as a result of participating?). The YAC recommended a mix of individual and focus group conversations, depending on participants’ ages and the particular program.

In addition, we intend to conduct individual conversations longitudinally, involving yearly interviews with the same nine participants over three years, regardless of whether they remain Satellite or not. This will allow us to explore why they remained or did not remain with Satellite, the accumulative impact of Satellite’s offerings (if applicable), looking for instances of continuity, change and growth over time [36]. A longitudinal approach also allows participants to reflect on changes since the previous conversation, anticipated future trajectories and whether and how Satellite is aligned with those narratives. From these conversations, we aim to capture critical moments and processes involved in change as well as what might have been needed to better support young people at different time points.

### Survey outcome measures and design

All young people who enter Satellite (approximately *n* = 150 each year) will be invited to participate in surveys on program entry, and then again six and 12 months later, using the number of programs they engage in as a variable. Using what we know from previous evaluations in this field [12, 16] and feedback on expected outcomes from the YAC (Table 2) we intend to use various questionnaires for the different age groups (See Table 3). Satellite have various outcomes in their charter, which relate to strengths-based areas that they hope to nurture in young people who are part of their programs. In consultation with Satellite staff and the YAC, the four most important outcomes were identified and valid, reliable measures were selected to align with these outcomes. Additionally, a similar group of Australian children/adolescents who self-identified as having caring responsibilities will be matched with young people who have participated in Satellite’s programs on demographics such as age and gender. The identified Longitudinal Study of Australian Children (LSAC) cohort will be used as a quasi “usual care” group on the outcomes of interest. Commencing in 2003, the LSAC is a national longitudinal study of data collected every two years on various child, parental and family characteristics that influence children’s development at different ages [37]. An a priori power analysis was conducted using G*Power version 3.1.9.7 software [38] to ascertain the minimum sample size required to test the study hypotheses. Results indicated that the required sample size to achieve 80% power for detecting a medium effect at a significance criterion of α = 0.05 will be 29 per group (intervention [Satellite participants] and usual care [LSAC])

**Table 3:** Outcome measures for children and youth

	Awareness/use of coping strategies		Reduced isolation/increase belonging	Mental wellbeing	Develop agency	Items from the Longitudinal Study of Australian Children [37]				
	Kids Coping Scale [46]	Coping Across Situations Questionnaire [47]	Children & Youth Resilience Measure [48]	Strengths & Difficulties Questionnaire [49]	Children’s Hope Scale [50]	Help seeking - support services	Help seeking - family	Mental wellbeing	Caring responsibilities	Belonging
Children (≤ 10 years)	X	-	X	-	X	-	-	-	-	-
Children (11–13 years)	-	X	X	X	X	-	-	-	-	-
Adolescents (14–17 years)	-	X	X	X	X	X^a^	X	X^a^	X^a^	X
Young adults (18+)		X	X	X	X	X^a^	X^a^	X^a^	X^a^	X^a^

Note. ^a^ Denotes questions that will be compared with responses from LSAC participants (i.e., control group who have not accessed services from the Satellite Foundation)

### Creative outputs

Across the various Satellite programs, young people are engaged in many different creative activities, including song writing and photovoice. All participants will be invited at program completion to share their creative outputs with a member of the evaluation team. This process will be very different from the interviews or questionnaire evaluation components. The way the young person wants to portray their creative work, and how they want to describe it will be up to them. Nonetheless, with the permission of the young person (and if under 16 years of age, the additional permission of their parent/caregiver), these artworks will be featured in the evaluation findings and reported as a means of describing the types of activities Satellite offers and the types of experiences and possible outcomes for the people involved [39]. Ideally, a YAC representative will be available to engage with the young person about their experiences of creating these outputs and the meaning they attribute to the pieces. The messages conveyed will be either via video or in text and photographs.

### Data analysis

#### Qualitative

Transcripts for the one-off conversations will be analysed within an inductive qualitative paradigm, using the six-step reflexive thematic process recommended by Braun and Clarke [40]. Thematic analysis is flexible to the format of the collected data and theoretically independent. Participants’ and researchers’ sociocultural interpretations are expected to influence the structure of themes. The analysis involves becoming familiar with the data by reading and re-reading each transcript, generating initial codes, searching for and then reviewing themes and then defining and naming themes. Intercoder consistency will be employed, whereby one member of the team will identify themes and follow this up with a group (evaluation team plus YAC) discussion of overlaps and divergences [41]. Specific data sets will be linked to particular programs and then compared and contrasted to see if there are overall patterns across programs or unique results to any particular offering.

Transcripts for the longitudinal conversations over three years will be managed using a longitudinal coding matrix template. A within-case analysis will initially be conducted with each interview set, then a cross-case analysis of patterns emerging over time across interviews [36].

#### Quantitative

The impact of Satellite on young people will be examined using the same outcome measures across the three time points using multi-level modelling (see Table 3). This will allow examination of whether the number of programs that young people enrol in over the 12 months results in different outcomes, as well as controlling for other variables such as age and gender. This model will also allow people to be entered into the analyses even if they do not complete the questionnaire at every time point (i.e., only complete the questionnaires at two of the three time points). Outcomes of interest will also be compared across groups (participants in the Satellite programs and the “usual care” (LSAC) participants) using repeated-measures MANOVA.

### Ethics

Working with young people, especially those living with adversity, requires several ethical considerations. Parental consent is typically needed for children and young people under the age of 16, though previously, some parents from these families have actively or inadvertently limited their children’s involvement in research [42]. For instance, previous research has found that some parents are reluctant for their children to discuss what they considered to be family problems to outsiders while others consider that the process may be too upsetting for their children [42]. Additionally, if parents are very unwell and/or hospitalised they will not be eligible to participate, given that the parent is not able to provide informed consent. In addition to parental consent for those aged under 16, we will also seek child assent for their involvement.

Age appropriate language will be used to ensure informed consent/assent. As per Reupert et al. [42],young people will be given the option of not being involved (even if their parent has given consent to their involvement), e.g., “You don’t have to be involved in this interview if you don’t want to be, no one will be angry with you”. They will be told not only about their right to withdraw, but also how they might do this (e.g. say they have changed their mind) [43]. As recommended by Spriggs [44], children and young people will be able to withdraw from the evaluation at any time up to when the results are written up in reports/publications. Children and young people will be assured that unless they are at risk themselves, or may harm someone else, no one outside of the evaluation team will have access to what they said.

Interviews will be conducted in a COVID safe and accessible way. In discussing their involvement with Satellite, children and young people may become upset or uncomfortable and the potential for this will be clearly communicated. Given this, the interviewer will be sensitive to the verbal and nonverbal cues of the children and young people interviewed, alert to the need to pause or end the interview and offer further assistance and/or professional support as needed. At the conclusion of each interview, the child or young person will be asked whether they had any concerns or require any additional support. They will be provided with organisations to contact if they need support at a later stage. While acknowledging the potentially distressing nature of the evaluation process for children and young people, we are simultaneously mindful of Gladstone’s et al. [13] argument that although vulnerable, these young people are often insightful and autonomous, and have a right to discuss their experiences, especially concerning services that purport to support their needs. It can be cathartic to be part of an evaluation project, as it gives young people and children an opportunity to reflect on their individual circumstances; it can also be empowering to be consulted in a genuine, authentic manner.

Ethics approval has been obtained from the Monash University Human Research Ethics Committee (MUHREC):project number 31,681.

### Findings and dissemination

Throughout the project, consistent with participatory methods, all the collected, de-identified results will be workshopped with the YAC to discuss program implications. Evaluation reports will be generated six times at six-monthly intervals over the three years with a final report submitted at the end of the project. In order to make the various evaluation reports accessible to various audiences, especially young people, findings will be presented in diverse ways, tailored to the needs of different audiences, in written form and/or through video, and infographics. Analysed data will be presented to the YAC and Satellite staff every six months over the three-year project, with subsequent recommendations made for program delivery and the evaluation design. If Satellite decides to make changes in a program, consideration will be made as to evaluation implications e.g. the addition of other measures, or additional interview questions. Thus, subsequent program changes will be monitored and reflected in the evaluation plan. The final evaluation report will be produced in multiple formats; one specifically with the funders as the intended audience, and another for the young people using accessible language, with both reports being publicly available. The YAC will be consulted as to the best ways of disseminating results to the community, with an option also of a public event with media invited at the end of the project.

Along with evaluation results, the number of participants who were involved in the various programs will be recorded, as there will likely be more who participate in the programs than participate in the evaluation. This information will include a breakdown of different demographic groups and identify those groups that Satellite have not yet been successful in attracting. For example, Maybery et al. [18] found it difficult to recruit young men into a similar early intervention program. Together with the YAC, strategies will be developed to target any missing groups into programs or efforts made to ascertain why specific demographic groups are not being recruited into Satellite programs. Referral sources will be reported, identifying those organisations referring young people to Satellite, and arguably more importantly, which services are not, which will allow for targeted messages to selective services.

## Discussion

In program evaluation, there is a need to balance the accountability needs of funders with the needs of organisations and their clients. Evaluations need to deliver rigorous, objective data that inform government funders and organisations whether the programs being delivered are making a positive difference to those they are intended. For evaluations involving young people, their involvement in the evaluation process is critical to ensure that findings are relevant and can be used to inform ongoing quality improvement, program development and decision making about the long-term offerings of programs.

There are many different ways young people may participate in program development and evaluation, ranging from being merely informed about programs through to active co-design opportunities where young people are equal partners in decision-making [20–22]. This study protocol offers an approach that provides multiple opportunities for different groups of young people to be involved, although it is at the collaborative level rather than full co-design. Nonetheless, through collaboration, involvement and consultation, we aim to provide opportunities to young people for empowerment and self and professional development while at the same time meeting the needs of government funders.

Despite the broad acknowledgement that it is critical to include end-user stakeholders in program development and evaluation, many participatory designs do not explicitly describe or reflect on the specific participatory processes employed [20]. LeRoux [45] described how some non-for-profit organisations spend disproportionate time addressing the needs of funding agencies at the expense of client-related activities, albeit in the interests of maintaining a funding flow to sustain their client-related work. This protocol provides one example of how to balance the needs of funding departments and still include the voices and experiences of young people, a process that might be used and adapted by the broader policy community.

## Conclusion

Data generated from this novel, mixed methods participatory evaluation will be used to further inform programs and services at Satellite for young people who have a parent or a sibling who experiences mental health challenges. The longitudinal interviews and the utilization of creative outputs have not previously been used in evaluations of this type and have the potential to shed new light on the change processes involved in similar programs. The participatory evaluation design and methodologies employed could be used by other services when conducting similar evaluations. When evaluating programs for young people, tension may exist between meeting the needs of funders and the needs and preferences of end users, especially young people. This protocol highlights how this tension might be addressed by using validated outcome measures and rigorous evaluation design alongside the provision of multiple opportunities and approaches for collaborating with young people about what is being evaluated and how.

## Data Availability

The datasets used and analysed during the current study will be available from the corresponding author on reasonable request.
